# Assessing the Potential Threat Landscape of a Proposed Reintroduction Site for Carnivores

**DOI:** 10.1371/journal.pone.0122782

**Published:** 2015-03-30

**Authors:** Samantha K. Page, Daniel M. Parker, Dean M. Peinke, Harriet T. Davies-Mostert

**Affiliations:** 1 Wildlife and Reserve Management Research Group, Zoology and Entomology Department, Rhodes University, Grahamstown, South Africa; 2 Endangered Wildlife Trust, Modderfontein, Johannesburg, South Africa; 3 Eastern Cape Parks and Tourism Agency, East London, South Africa; University of Florida, UNITED STATES

## Abstract

This study provides a framework to assess the feasibility of reintroducing carnivores into an area, using African wild dogs (*Lycaon pictus*) as an example. The Great Fish River Nature Reserve in the Eastern Cape Province, South Africa, has been identified as a potential reserve to reintroduce wild dogs, and we applied this framework to provide a threat assessment of the surrounding area to determine potential levels of human-wildlife conflict. Although 56% of neighbouring landowners and local communities were positive about a wild dog reintroduction, data collected from questionnaire surveys revealed that human-wild dog conflict is a potential threat to wild dog survival in the area. Additional potential threats include diseases, snaring, poaching and hunting wild dogs for the use of traditional medicine. A threat index was developed to establish which properties harboured the greatest threats to wild dogs. This index was significantly influenced by the respondent’s first language (isiXhosa had more positive indices), education level (poorer education was synonymous with more positive threat indices), land use (wildlife ranching being the most negative) and land tenure (community respondents had more positive indices than private landowners). Although threats are present, they can be effectively mitigated through strategies such as carnivore education programs, vaccination campaigns and anti-snare patrols to promote a successful reintroduction of this endangered canid.

## Introduction

As a direct consequence of globally declining animal populations, conservation and management of small, disjunct populations has become inevitable [1]. Reintroductions have become an important conservation strategy to support population recovery [1,2] and to re-establish a species within its historical range after being locally extirpated [1]. Evaluation of the suitability of proposed reintroduction sites must be conducted prior to any reintroduction effort [3,4]. The proposed site should ideally fall within the historical range of the species, the population must be sustainable for the foreseeable future, and there should be sufficient capacity for the site to sustain the diet of the reintroduced species [2,3]. An additional, and crucial, part of reintroduction planning is to assess the potential threats in and around the reintroduction site. Prefeasibility assessment is used to determine whether a site can sustain a reintroduced population and includes determining whether the previous causes for extirpation still remain a threat.

The global population of endangered African wild dogs (*Lycaon pictus*) is currently estimated to be around 6600 individuals [5,6], with approximately 420 occurring in South Africa [7], making them the most endangered carnivore in southern Africa [5,6]. Viable wild dog populations (defined as a self-sustaining population where packs survive without human intervention [8,9]) are limited to approximately six of the 39 countries where they historically occurred [10]. The Kruger National Park is considered to have the only viable population of wild dogs in South Africa [8]. As no additional protected areas in South Africa are large enough to sustain such a population, recent conservation efforts have concentrated on establishing a managed metapopulation, where subpopulations of wild dogs are managed in several small (≤ 1000 km^2^), geographically isolated conservation areas as a single, linked metapopulation [11]. This approach involves reintroductions and the periodic translocation of individuals among packs from different reserves to mimic natural dispersal and promote gene flow [12]. To date, the metapopulation approach has been successful in South Africa, with high survival rates of the released packs and their offspring [13–15]. Through the duration of this study there were between 150 and 156 wild dogs in roughly 19 packs that were distributed across eight metapopulation reserves in South Africa [7]. However, increasing the number of metapopulation reserves remains a priority [8,14]. The Great Fish River Nature Reserve (GFRNR) in the Eastern Cape, South Africa, has been identified as a potentially suitable site for a wild dog population to be reintroduced [7,8].

Wild dogs were extirpated from the Eastern Cape by the mid-1900s mainly due to persecution by livestock farmers [16,17]. Given that wild dogs are one of the widest-ranging African carnivores [9,18], and are difficult to contain in fenced areas [10,18], conflict with livestock and game farmers (if present) must be assessed and managed [9,19,20]. Thus, assessing the potential threats to wild dog survival on unprotected land adjoining the GFRNR is imperative. These threats include conflict with surrounding farmers and neighbouring communities [8,13,16], poaching, snaring [9–11] and diseases [10].

Our study measured the potential for human-wild dog conflict, gauged the potential for disease transmission (especially rabies and canine distemper virus) from domestic dogs (*Canis familiaris*), and evaluated the prevalence of snaring and wildlife poaching on both private and communal properties adjacent to the GFRNR. We also developed a global approach for assessing the potential threat landscape that could readily be adapted for other reintroduced carnivores. This approach involves surveying all stakeholders in the vicinity of the reintroduction site. As these stakeholders will be potentially impacted by the reintroduction, it is important to understand their concerns and their behaviour following reintroduction. After an area-specific questionnaire has been completed, a threat index can provide a quantitative representation of the potential threats to the reintroduced carnivore and identify potential mitigation measures to reduce these threats (such as compensation schemes, improving fencing, etc.). This approach evaluates the potential for known threats to wild dogs [6,10,14,20] that could impact the reintroduction. The index created converts categorical information into a continuous measure, useful for obtaining a landscape level view of risks [21]. While our study uses this approach for wild dogs, this technique could also be widely adapted for other carnivore species.

## Methods

### Study Area

**Fig 1:**
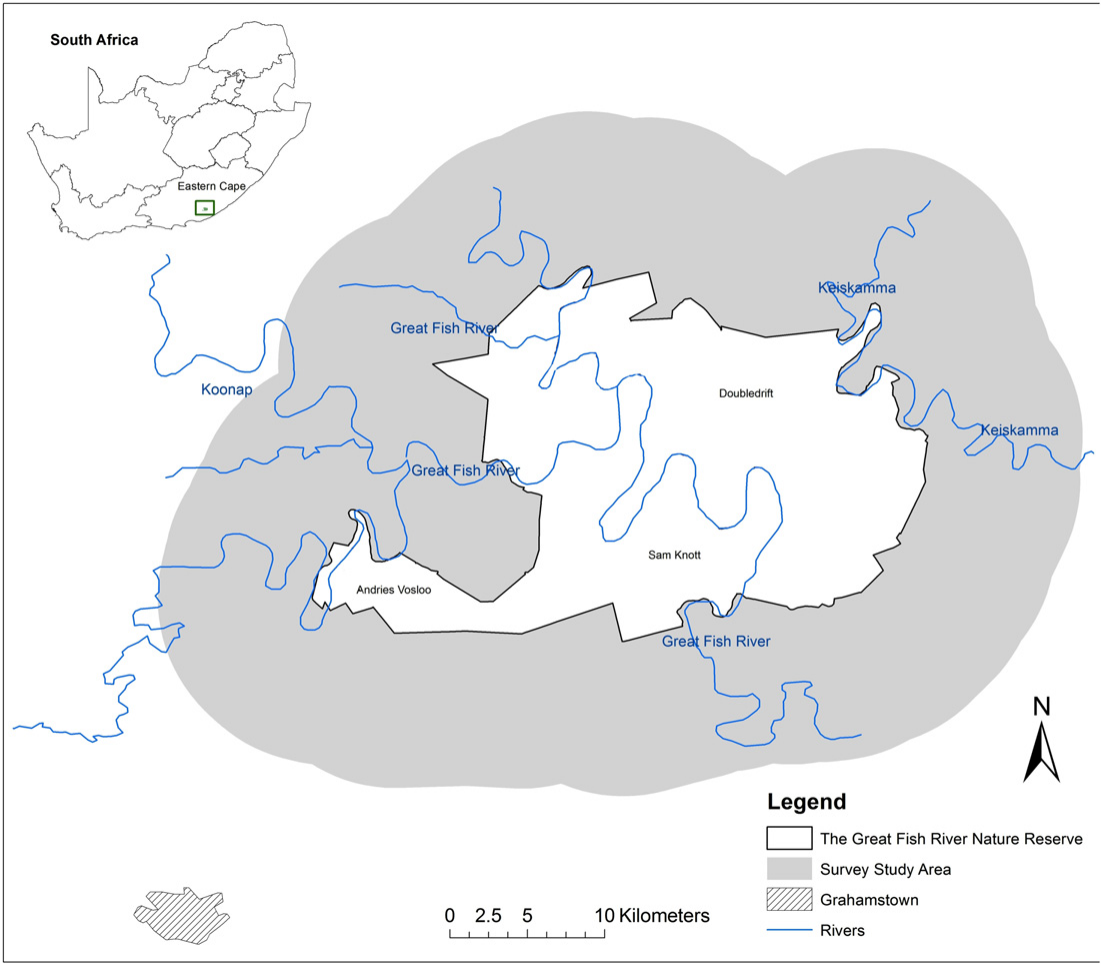
The Great Fish River Nature Reserve and the study area within South Africa.

The GFRNR is situated in the Fish River Valley, Eastern Cape, South Africa (Fig 1; [22]), approximately 40 km northeast of Grahamstown and 32 km south of Alice [23,24]. The reserve covers an area of 429 km^2^ and is characterized by dense, semi-succulent thicket with considerable variations in topography and elevation [23,25]. Communal land is situated to the north and east of the reserve, whereas the areas to the south and west are generally made up of private properties. Communal areas are used largely for small-scale stock ranching and subsistence crop farming. Private land uses include international trophy hunting, eco-tourism and stock ranching. Notwithstanding differences in land use, the areas surrounding the GFRNR are generally characterised by a low population density (72 people/km^2^ in communal land and 7 people/km^2^ for privately owned land), and high levels of unemployment (> 50%), especially in the communal areas [23,25].

### Interview methods and questionnaire design

To obtain information on the potential threats to wild dogs outside the GFRNR, landowners within 12 km buffer of the reserve boundary were interviewed using structured questionnaires [26]. Clearance to conduct the work was granted by the Eastern Cape Parks and Tourism Agency (research agreement number RA0143). The width of this buffer zone was based upon the mean daily distance (defined as one 24 hour period) moved by a pack of wild dogs (10 km; [27]) and their average home range size (218km^2^; [27–29]). Daily movement wasused in this calculation because it was assumed to be the minimum amount of time required for reserve managers to recapture any dispersing/escaping animals. The average home range size of wild dogs in eastern southern Africa is 218 km^2^; 14.76 km being the approximate diameter [29]. Thus, taking the average of the mean daily distance moved (10 km) and the home range diameter (14.76 km), the landowners within 12 km of the GFRNR were assumed to be the most likely affected by any dispersing wild dogs.

Interviews (n = 128) were conducted in person between May and September 2013 in the respondent’s first language to ensure maximum completion and understanding [30,31]. In the communal areas, workshops were held to introduce the project to community members, prior to the interviews being done. These workshops were held in isiXhosa and were necessary to secure the participation of the various communities and to provide essential context. Rural community respondents (n = 91) typically spoke isiXhosa, while the first language of most private landowners surrounding the GFRNR (n = 37) was English [26]. Private landowners were interviewed one-on-one on an ad hoc basis [26]. The most senior person in charge of the property was interviewed, as these respondents were selected as they would be the decision makers on the property. Prospective respondents all gave prior informed consent and had the right to refuse being interviewed. Interviews took ~30 minutes to complete.

### Threat index

A threat index, generated from 16 questions in the questionnaire [26], was derived to quantify the potential threats to wild dogs on properties outside the GFRNR [32]. These threats included poaching (defined as the illegal direct hunting of a species), the potential for persecution through conflict, the presence of unvaccinated domestic dogs and snaring (indirect and unspecific poaching activities such as setting wire traps). Answers to trichotomous questions (yes/no/maybe) were assigned values in order to generate index scores [21,31,33]. Index scores were calculated by allocating values of between 1 and -1 to the questions according to a positive (1), neutral (0) or negative (-1) response [21,31]. The value for each index, for each of the respondents, was calculated as the sum of the scores of all 16 questions [21]. For example, the questionnaire asked: “I would harm wild dogs if they appear on my land”. A score of +1 was given if the respondent answered no (as it indicates a positive attitude and therefore lower threat), 0 if they were unsure and -1 if they answered yes (as it indicates a negative attitude and increased the potential threat to wild dogs). The maximum value that could be achieved for the threat index was 16, which indicated no or few threats in the area, while -16 was the most negative and indicated an area or property with many potential threats to wild dogs [26].

### The effects of demographic and property characteristics on the threat index

Multi-model analysis was used to determine the effects of five demographic variables (age, education level, language, gender and fear of wild dogs) and four property variables (land use, owning livestock/game, previous problems with predators and land tenure) on the threat index of respondents. Using Akaike’s Information Criterion adjusted for small sample size (AICc), demographic and property variables were incorporated into a best subsets model selection procedure [33]. The aim of this model selection approach was to identify which variables, when tested in combination, best predicted the threat index [34–38]. Prior to analysis, Spearman’s rank correlation matrices were created to confirm that none of the continuous predictor variables were inter-correlated [21]. Each model was constructed with between one and five variables, giving a combination of 31 potential demographic models and 15 potential property variable models that could explain the threat index.

Models were ranked in order of parsimony using AICc values [20,33]. Akaike weights (w*i*) were used to indicate the strength of evidence for a model, with higher values indicating that a model was relatively more important than the other models and therefore more likely to explain the variability in the data [34,35,39]. In addition to the model-building, impact factors were calculated in order to determine a variable’s relative importance in influencing the threat index [36]. Impact factors of > 0.80 were interpreted as strong evidence for the role of the predictor variable in shaping the threat index [36]. The statistical significance was set at p < 0.05 and all data were analyzed using Statistica 11.0 software (StatSoft inc. Tulsa, OK, USA; [36]).

## Results

### Respondent characteristics

Of the 128 respondents, 79% were males and the mean age of all respondents was 47 years ± SD 14.66 (range: 21–79). The relatively high proportion of older males in the sample can be attributed to the fact that in most cases the heads of the households and thus the primary decision makers in each household were interviewed. Private landowners made up 29% of the respondents and the balance was made up of communal landowners. IsiXhosa speakers made up 72% of surveyed respondents (99% of respondents in the communal areas), 20% were English speakers and 8% were Afrikaans speakers. Tertiary education was the highest level of education achieved by 14% of respondents. Most respondents (60%) only had high school education, 23% of respondents only had primary school education and the remaining 3% had no form of schooling.

Stock ranching was the dominant land use (67% of properties) in the area and the second most important economic activity was wildlife ranching (12%; for the purpose of hunting, live game sales and ecotourism). Both subsistence stock ranching and subsistence crop farming were more prevalent in the communal areas than on the private land. Crop farming was done by 7% of respondents and 14% of properties had no land use. The size of private properties ranged from 24 to 20000 ha (mean: 2 255 ± SD 136 ha).

Almost all respondents (94%) agreed that wild dogs should be conserved. However, only 56% were in favour of the reintroduction of wild dogs to GFRNR and 20% were against it (the remaining 24% were unsure). Perceived threat to livestock and/or game was the main reason given for opposing the reintroduction (Table 1). Livestock was kept by 87% of respondents, with cattle (*Bos primigenius*), goats (*Capra aegagrus hircus*) and sheep (*Ovis aries*) being the most prevalent animals. Personal fear of wild dogs was another important reason cited by respondents against the reintroduction (Table 1). Respondents in favour of the reintroduction gave six statements justifying their opinion (Table 1). The main motivation for supporting the reintroduction was the potential ensuing benefits including increased job opportunities and ecotourism contributions to the local economy (Table 1).

**Table 1:** Reasons given by respondents to justify willingness/unwillingness to reintroduce wild dogs to the Great Fish River Nature Reserve.

		Number of responses	
Attitude towards wild dog	Reasons given to justify (un)willingness to reintroduce wild dogs	Private	Communal Areas
**Positive**	Wild dogs are endangered and they need to be conserved	5	6
	Love them	3	2
	They are indigenous to the area	4	
	Will bring benefits (improve local economy, increase tourism, jobs)		21
	Not dangerous to human life		8
	To see them and have children see them		5
**Negative**	Scared of them		3
	Danger to livestock	2	17
	Threat to natural game inside and outside the reserve	2	
	They will break out of the reserve	1	

### Current perceptions of predators and the potential threats for wild dogs

When asked what their reaction would be to predators on their land, 58% respondents gave positive responses. Most respondents (48%) said that they would call the reserve managers to remove the predators, and 9% said that they would leave the animal(s) alone. However, 37% of respondents had more negative responses, including killing the predator (31%), and chasing it away (6%).

When asked about fear towards wild dogs, 39% of respondents expressed fear for their own lives and/or livestock and/or game. Wild dogs would be harmed by 9% of respondents if they appeared on their land. If wild dogs killed any of their livestock or game, this figure increased to 22%. Some form of predator control (including lethal techniques such as hunting, setting snares and poisoning, and non-lethal measures such as setting traps and using guard dogs) was already employed by 76% of respondents. Predators (including black-backed jackal *Canis mesomelas*, caracal *Caracal caracal* and brown hyena *Hyaena brunnea*) had previously been killed by 80% of respondents. Most (96%) of those who had not heard of wild dogs were communal area respondents. Compensation was expected by 85% of respondents if wild dogs were to kill any of their livestock or game.

### The threat index

**Fig 2:**
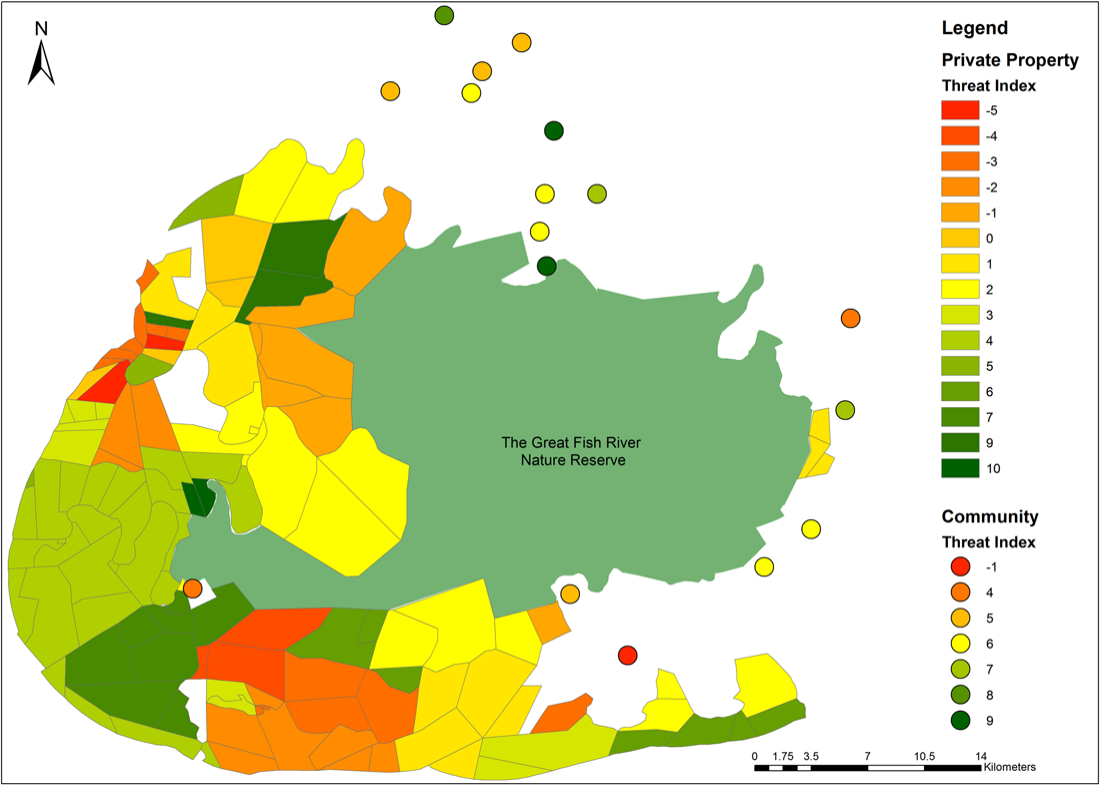
The threat index scores of the communal area (represented as an average index score of the respondents for each community) and private properties surrounding the Great Fish River Nature Reserve. (Reds and oranges indicate higher threat areas while darker greens indicate low threat areas).

The mean threat index for wild dogs was 4.00 ± SD 4.17 (range: -5 to 13). Private landowners had a mean threat index of 1.59 ± 3.89 (range: -5 to 10), while communal area members had a mean index of 5.46 ± 3.75 (range: -5 to 13). The most negative threat index was -5, indicating several potentially high threat areas for wild dogs. However, the most positive index achieved was 13, indicating that several low risk areas also exist (Fig 2). As respondents from the community interviews do not have specific property boundaries (unlike private landowners), they could not be visually represented individually in Fig 2. Rather, communities were illustrated by averaging the scores achieved by their represented respondents, therefore figures shown are an average score of respondents for that community.

Generally, while still positive, most private properties adjacent to the reserve had low threat index values (Fig 2). The threat indices of these properties were influenced by poaching, snaring and the presence of unvaccinated domestic dogs.

**Fig 3:**
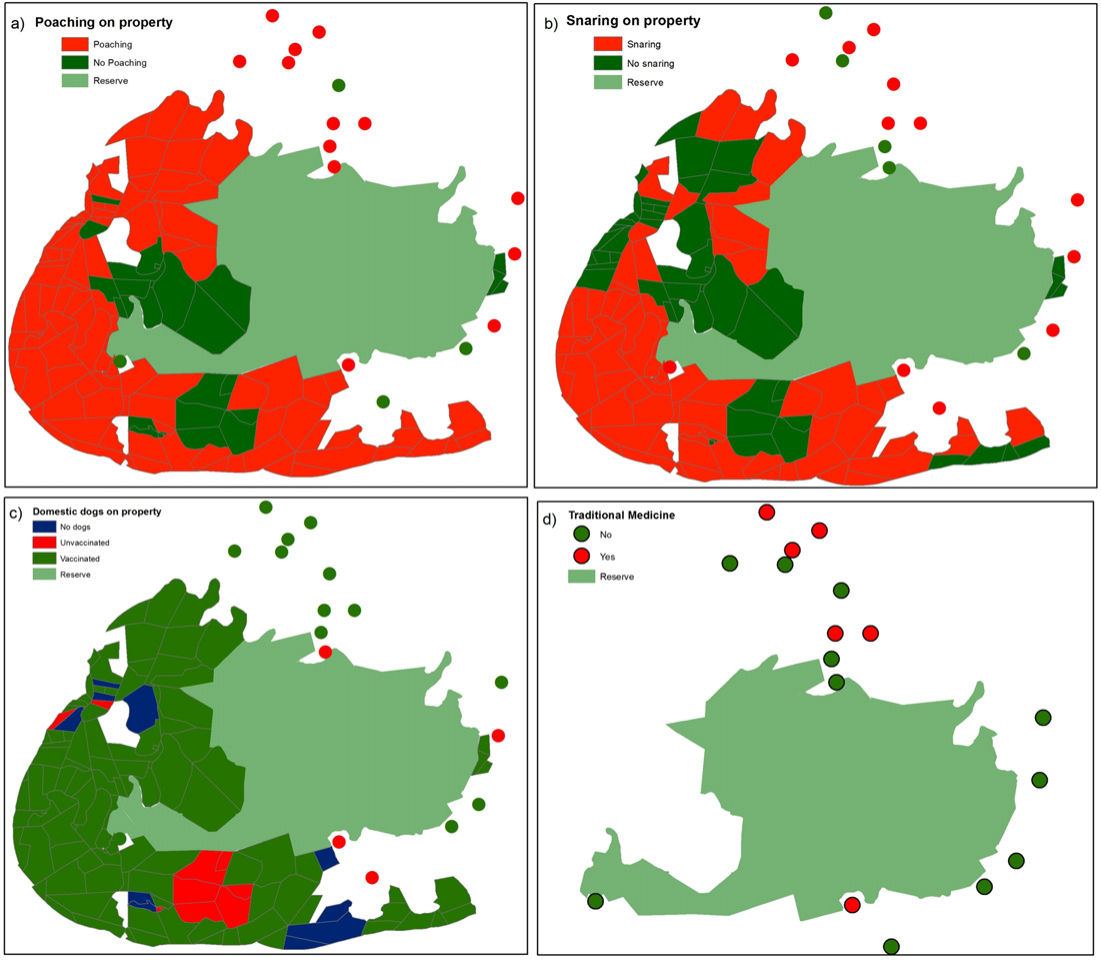
Threats to wild dogs around the Great Fish River Nature Reserve. A) Poaching, B) Snaring, C) Domestic dogs and D) Wild dogs used for traditional medicine.

Most respondents believed that poaching was a severe problem around the reserve, especially on the private properties (Fig 3a). Although poaching was experienced by 80% of respondents, and 79% of respondents regularly patrolled their fence lines to search for wire snares, only 87% removed them while the others left them in place. In the year preceding our interviews, 73% of private landowners had experienced some form of poaching, and 38% of communal area respondents had experienced poaching on their land.

Domestic dogs occurred on 81% of private properties, 88% of which were vaccinated against canine distemper and rabies (Fig 3c). Of the private property respondents who owned dogs, there were a total of 126 dogs (13 unvaccinated on four properties; Fig 3c). Domestic dogs were owned by 82% of communal area respondents and 85% were vaccinated (Fig 3c). At least 179 dogs were owned by the 75 communal area respondents; at least 27 of these dogs were unvaccinated (in four communities; Fig 3c). Most respondents used their dogs for guarding livestock while three communal area respondents used their dogs for hunting.

None of the respondents from private properties reported any traditional uses for wild dogs (Fig 3d). However, 10 respondents from six communities (8% of respondents) stated that they knew of traditional uses for wild dogs (Fig 3d). According to one respondent, “the smoke of burning wild dog fur makes someone sleep” while another stated that “traditional healers use them in their traditional attire and that they are used for many medicinal purposes”.

### Relationship between demographic variables and threat index

Two models (which included age, gender, first language and fear towards wild dogs) had the lowest ΔAICc values and were thus the most suitable models for explaining the observed variation in the threat index (Table 2).

**Table 2:** The Akaike information criterion (AICc) values for regression models investigating the effects of human socio-demographic variables on the threat index of respondents (n = 128).

	Variable 1	Variable 2	Variable 3	Variable 4	Variable 5	AICc	ΔAICc	wt
1	Age	Gender	Language			688.33	0	0.42
2	Age	Gender	Language	Fear		689.24	0.91	0.27
3	Age	Language				690.85	2.52	0.12
4	Age	Language	Fear			691.71	3.38	0.08
5	Age	Gender	Education	Language		693.8	5.47	0.03
6	Gender	Language				693.99	5.67	0.02
7	Age	Gender	Education	Language	Fear	694.73	6.4	0.02
8	Language					695.41	7.08	0.01
9	Age	Education	Language			695.67	7.34	0.01
10	Gender	Language	Fear			696.07	7.75	0.01
11	Age	Education	Language	Fear		696.62	8.29	0.01
12	Language	Fear				697.11	8.78	0.01
13	Gender	Education	Language			698.2	9.87	0
14	Gender	Education	Language	Fear		699.73	11.4	0
15	Education	Language				700.1	11.77	0
16	Education	Language	Fear			701.25	12.92	0
17	Age	Gender	Education			707.1	18.78	0
18	Gender	Education				707.24	18.91	0
19	Age	Gender				707.63	19.3	0
20	Age	Gender	Education	Fear		708.37	20.04	0
21	Age	Gender	Fear			708.88	20.55	0
22	Gender	Education	Fear			709.35	21.02	0
23	Gender					709.94	21.61	0
24	Education					711.26	22.94	0
25	Age	Education				711.84	23.51	0
26	Gender	Fear				712.52	24.19	0
27	Age					712.82	24.5	0
28	Education	Fear				713.96	25.63	0
29	Age	Education	Fear			714.14	25.82	0
30	Age	Fear				715.61	27.28	0
31	Fear					718.37	30.04	0

**Fig 4:**
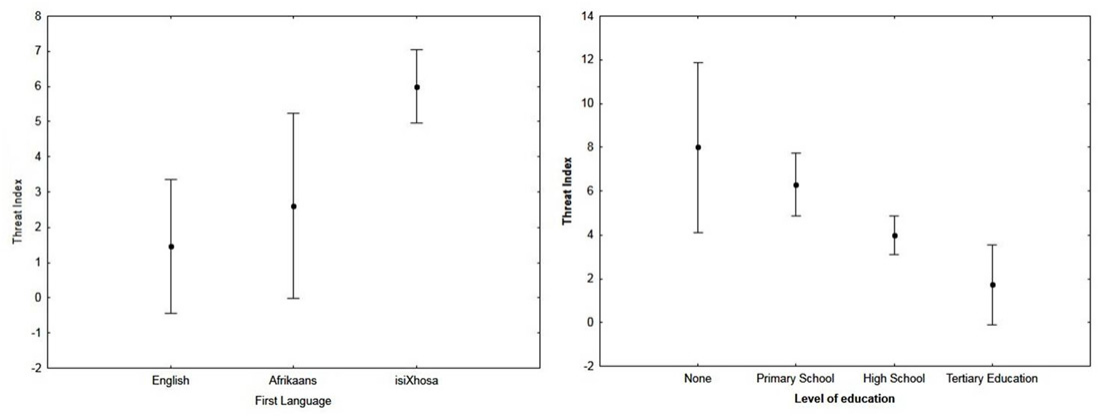
The significant relationships between the first language of a respondent and their threat index (F_(2; 118)_ = 13.87; p < 0.05) and the level of education of respondent and threat index (F_(3; 123)_ = 3.53; p < 0.05).

First language was the most important individual predictor variable for the threat index (impact score of 1.00; Table 3). The effect of language on the threat index was significant (F_(2; 118)_ = 13.87; p < 0.05). IsiXhosa first language speakers had the most positive threat index (6.11 ± 3.76), Afrikaans speakers received an index of 2.30 ± 3.86 and English speakers had the poorest index (1.35 ± 4.02; Fig 4). The difference in threat indices between isiXhosa and English speakers was significant (p < 0.05). The threat index of respondents generally became more negative with increasing education levels (F_(3;123)_ = 3.53; p < 0.05; Fig 4). Respondents with tertiary education received the poorest index (1.72 ± 3.85), respondents who had completed high school received an index of 4.00 ± 4.00, respondents who only completed primary school had an index of 6.30 ± 3.49, and respondents with no education received the most positive index (8.00 ± 4.08; Fig 4). While the level of education significantly influenced a respondent’s threat index, it had the lowest impact score of the five predictor variables (0.07; Table 3). In addition, threat indices became more positive with increasing age, but they were generally randomly distributed across age groups and explained 1% of the variation in the threat index (F_(1; 128)_ = 3.58; p > 0.05; r^2^ = 0.01). The mean threat index was more positive for females than males, scoring 5.26 ± 3.59 and 4.10 ± 4.29 respectively (F_(1; 126)_ = 1.66; p < 0.05). Fear towards wild dogs did not have a significant effect on the threat indices of respondents towards wild dogs (F_(1; 126)_ = 3.04; p > 0.05). Respondents who feared wild dogs had a mean index of 4.23 ± 1.15 while those respondents who did not fear wild dogs had an index of 6.00 ± 2.31.

**Table 3:** The individual Akaike weights (impact factors) for the human demographic variables predicting the threat index of respondents towards wild dogs in the Great Fish River Nature Reserve.

Variable	Impact Factor
First language	1
Age	0.94
Gender	0.77
Fear towards wild dogs	0.38
Level of education	0.07

### Relationship between property variables and threat index

Two models (which included land tenure (community or private), land use and previous problems with predators) were the most suitable models for explaining the variation in threat index (Table 4).

**Table 4:** The Akaike information criterion (AICc) values for regression models investigating the effects of property variables on the threat index of respondents.

	Variable 1	Variable 2	Variable 3	Variable 4	AICc	ΔAICc	wt
1	Land tenure	Land use	Problems with preds.		697.29	0.00	0.39
2	Land tenure	Land use			698.57	1.28	0.20
3	Land tenure	Land use	Own livestock or game	Problems with preds.	698.79	1.50	0.18
4	Land tenure	Land use	Own livestock or game		700.04	2.75	0.10
5	Land tenure	Problems with preds.			701.34	4.05	0.05
6	Land tenure				701.98	4.69	0.04
7	Land tenure	Own livestock or game	Problems with preds.		703.28	5.99	0.02
8	Land tenure	Own livestock or game			703.55	6.25	0.02
9	Land use	Problems with preds.			711.56	14.27	0.00
10	Land use	Own livestock or game	Problems with preds.		713.52	16.23	0.00
11	Land use				715.33	18.03	0.00
12	Land use	Own livestock or game			717.08	19.79	0.00
13	Problems with preds.				723.30	26.00	0.00
14	Own livestock or game	Problems with preds.			725.29	28.00	0.00
15	Own livestock or game				726.99	29.70	0.00

**Fig 5:**
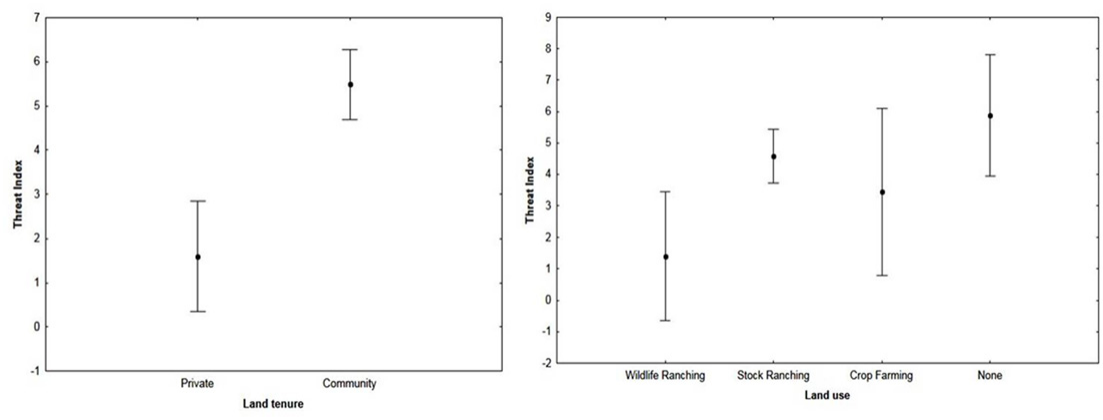
The relationships between the threat index of a respondent and their properties land use (F_(3; 123)_ = 3.75; p < 0.05) and land tenure (F_(1;116)_ = 16.44; p < 0.05) was found to be significant.

Land tenure was the best individual predictor variable for the threat index and had a significant effect on the respondents’ index (impact score of 1.00; F_(1; 116)_ = 16.44; p < 0.05; Table 5). Private landowners had a mean index of 1.59 ± 3.89, while communal area respondents had a mean index of 5.46 ± 3.75 (Fig 5). It was expected that if a property carried livestock or game, the respondents would have had a more negative threat index than properties without these animals. However, irrespective of the presence of livestock or game, threat indices were similar and the effect was insignificant (F_(1; 116)_ = 0.05; p > 0.05). Although the relationship between these variables was insignificant, their impact scores were high (Table 5). It was also found that if respondents experienced problems with poaching, they tended to have more negative threat indices than those who had not experienced poaching (F_(1; 126)_ = 19.82; p < 0.05).

**Table 5:** The individual Akaike weights (impact factors) for the property variables predicting the threat index of respondents towards wild dogs in the Great Fish River Nature Reserve.

Variable	Impact Factors
Land tenure	1.00
Land use	0.87
Previous problems with predators on property	0.64
Own domestic livestock or game	0.32

Land use was the second-best predictor of the threat index (an impact score of 0.87; Table 5). The effect of land use on the respondent’s threat index was significant (F_(3; 123)_ = 3.75; p < 0.05). Properties that had no land use had the most positive index (5.88 ± 4.33), followed by stock ranching properties (4.58 ± 4.03; Fig 5). Crop farming properties had a threat index of 3.88 ± 3.72, while wildlife ranching properties had the most negative threat index (1.21 ± 3.79; Fig 5). There was a significant difference in threat indices between wildlife ranching properties and those properties with no land use (p < 0.05).

## Discussion

Persecution by landowners on unprotected land is likely the most critical potential threat facing wild dogs following a reintroduction into GFRNR [20,40]. Persecution arises from fear of attacks on people and competition between landowners and the carnivore over resources [19,20]. Resources, including space and animals (livestock or game), are often limited, have an economic value to the owner [20]. While landowner tolerance towards wild dogs was generally positive (56%), 9% of respondents said that they would harm wild dogs and this increased to 22% if wild dogs killed any livestock or game. This attitude towards predators is often influenced by the demographic variables of the respondent.

Education level is often found to be a significant predictor of human tolerance towards predators [31,41]. Higher education is believed to lead to increased understanding of the ecological role of carnivores and with that, an increased acceptance and tolerance of predators [32]. In contrast to other studies [30,42,43], respondents in our study with primary school education or less had the most positive threat indices. This could possibly be because most of these respondents had not heard of wild dogs before the interviews were conducted. The 49 community respondents that had not heard of wild dogs before the study had a mean index of 5.73 ± 3.84, while the 42 community respondents who had heard of wild dogs had a mean index of 5.14 ± 3.67. Therefore, it is possible that those who had not heard of them had no prior misconceptions about wild dogs and were then more receptive to the potential reintroduction [13]. However, it is crucial to consider that the largest concern when using questionnaires is that of response bias [13]. This is a type of cognitive bias where respondents answer questions according to how they think the interviewer wants them to answer rather than according to their true beliefs [13]. This problem may have occurred with community questionnaires and this may have led to slightly exaggerated results where threats seem less prevalent than they are in reality. This might explain how poorer educated respondents from the community had more positive scores. However, we are confident with the accuracy and honesty of our results, as prior to the interviews respondents were assured that their answers would remain confidential and that there would be no negative ramifications for their responses. Significantly, most respondents felt comfortable, even admitting to being involved in illegal activity in the area.

Language significantly influenced the threat scores of our respondents. IsiXhosa speakers had the most positive threat index, followed by Afrikaans and English speakers respectively. As language can be used as a proxy for culture, it is apparent that the culture of the respondents influenced their attitudes towards a wild dog reintroduction [41,44]. English and Afrikaans individuals, who have a long tradition of commercial livestock farming in South Africa [41], tended to be less tolerant of predators on their properties. This may explain their more negative threat indices [20,45]. By contrast, isiXhosa individuals generally have an increased sense of interconnectedness with nature, as biodiversity is linked to strong nature-based religious beliefs in isiXhosa culture [44]. Thus, the isiXhosa communities surrounding the GFRNR may have a more innate desire to conserve biodiversity and their natural surroundings [44], which bodes well for a wild dog reintroduction. This argument probably also explains why respondents on private land (generally owned by English and Afrikaans speakers) had more negative threat indices than communal area respondents.

Respondents from properties that did not have domestic livestock or wild game had the most positive threat indices, as they were less likely to incur any financial losses from wild dog depredation [20]. Wildlife and stock ranchers received the poorest index scores (1.21 and 4.58, respectively). We expected stock farmers to have more negative indices than wildlife ranchers, as livestock farming is often less profitable than game ranching and thus the financial impact of predation can be higher [20,46]. However, livestock farmers in our study may have been more positive as predation on livestock can be prevented by using effective husbandry techniques (such as stop collars, guard dogs or livestock camps [47]).

Wild dogs have medicinal uses in many African cultures, and this could become a significant threat to the long-term survival of wild dogs in the GFRNR [48]. Ten communal area respondents in our study reported medicinal uses for wild dog products. Thus, there is the possibility that deliberate snaring and poaching of wild dogs may occur. As wild dogs occur in small populations, they are vulnerable to even small impacts such as deliberate killing. Therefore, dispelling common misconceptions of wild dogs, including their threats to humans and medicinal uses, addressing concerns of fear and monetary losses, and encouraging increased co-operation with reserve staff is vital [42,45].

The risk of disease transmission from domestic dogs is a potential threat to reintroduced wild dogs [49,50]. Wild dog populations that are small, isolated and occur close to human settlements (as the GFRNR population would be) are most at risk from disease outbreaks [48]. Wild dogs are known to be susceptible to diseases, particularly rabies and canine distemper virus [12,51,52]. The interaction between unvaccinated domestic dogs on adjacent properties and wild dogs within the reserve may increase the potential for disease transmission. Four communities, all located near (< 5 km) the reserve, had dogs that were unvaccinated against rabies or canine distemper. As the community members often allow their dogs to roam freely, it is likely that there may be interaction between these unvaccinated dogs and reintroduced wild dogs. Therefore, campaigns to vaccinate all domestic dogs in the vicinity of the GFRNR should be conducted prior to any wild dog reintroduction. Research by Fitzpatrick *et al*., [53] found that annual dog vaccination campaigns that achieve a vaccination target of 70% will control disease transmission with a high level of certainty. Evidently, 88% of domestic dogs in the buffer zone were vaccinated and therefore we do not consider disease transmission to be an imminent threat.

To promote a successful reintroduction and limit the number of anthropogenic wild dog mortalities, it is fundamental that reserve management implement mitigation strategies. These could include the implementation of a compensation scheme to reduce the potential conflict between wild dogs and landowners [42,54]. A compensation scheme, if implemented correctly and effectively, will enable the landowners to overcome losses and anger to prevent retaliation towards predators [42,55, 56]. However, compensation schemes present many challenges (e.g. lack of evidence to justify payout, lack of funding) and this approach needs to be carefully planned before implementation [42,55]. To further mitigate and reduce wild dog mortality, wild dogs can be fitted with anti-snare collars [57]. The purpose of this collar is to trap the wire snare in the rivets on the collar, thus protecting the neck of the dog [57]. Data from our interviews suggest that potential threats to wild dog survival do exist in the area immediately surrounding the GFRNR. However, the implementation of the strategic mitigation efforts described above prior to a reintroduction, can promote the long-term survival of wild dogs in the reserve. The vision of the South African National Action Plan for African Wild Dogs is to “secure wild dog populations within a matrix of land uses that contribute to ecosystem integrity, which coexist with, and are valued by the people of South Africa” [10]. Reintroducing wild dogs into the GFRNR will promote this vision and increase the overall genetic diversity and demographic resilience of the managed wild dog metapopulation. Significantly, the GFRNR can serve as a platform from which to educate and create awareness for this endangered carnivore.

In conclusion, the threat index we developed for the GFRNR provides a useful overview of the presence and distribution of potential threats to wild dogs at this possible reintroduction site. Our approach allows assessment of the feasibility of introduction and exposes geographic areas of concern. These areas can be targeted for the implementation of mitigation strategies to promote a successful reintroduction. We envisage that the threat index could be further refined by weighting individual threats to even more effectively prioritize mitigation action and that modifying these weights and the questionnaire for other large carnivores will make this a broadly applicable and useful tool for carnivore reintroductions around the world.
